# The Prevalence of Developmental Enamel Defects in Israeli Children and Its Association with Perinatal Conditions: A Cross-Sectional Study

**DOI:** 10.3390/children10050903

**Published:** 2023-05-19

**Authors:** Gisela Berenstein Ajzman, Nurit Dagon, Rabea Iraqi, Sigalit Blumer, Shada Fadela

**Affiliations:** Department of Pediatric Dentistry, The Maurice and Gabriela Goldschleger School of Dental Medicine, Tel Aviv University, Tel Aviv 6997801, Israel; jagisashyiali@gmail.com (G.B.A.); nurit.dagon@gmail.com (N.D.); r1be3i@gmail.com (R.I.); shadafadela@gmail.com (S.F.)

**Keywords:** child, molar hypomineralization, diagnosis, etiology, prevalence, risk factors

## Abstract

Molar incisor hypomineralization (MIH) and deciduous molar hypomineralization (DMH) affect the first permanent molars and second primary molars, respectively, causing a greater dental treatment burden and worse oral health quality of life among affected children. We assessed the prevalence and risk factors of MIH and DMH among 1209 children aged 3–13 years who attended a university dental clinic in Israel in 2019–2020. Clinical examinations were conducted to assess the presence of DMH and MIH. Potential etiological factors of MIH and DMH, including demographics, the mother’s perinatal health, and the child’s medical history during the first three years of life, were retrieved using a questionnaire. To examine the associations between the demographic and clinical variables and the prevalence of MIH and DMH, continuous variables were tested using the Kruskal–Wallis test with Bonferroni corrections. Categorical variables were analyzed by chi-squared test. Multivariate logistic regression was conducted to evaluate which of the significant variables found in the univariate analysis could predict a diagnosis of both MIH and DMH. The prevalence of MIH and DMH was 10.3% and 6.0%, respectively. Age ≥ 5 years, taking medications during pregnancy and severe lesions were associated with a greater risk for a diagnosis of DMH + MIH. Multivariate logistic regression with adjustment for age showed that the severity of hypomineralization was positively and significantly associated with a diagnosis of MIH + DMH with an odds ratio of 4.18 (95% confidence interval 1.26–17.16), *p* = 0.03. MIH should be diagnosed and monitored in young children to prevent further deterioration. Moreover, a preventive and restorative program for MIH should be established.

## 1. Introduction

The first permanent molars begin to develop in the fetus during the fourth month of gestation, and their mineralization first occurs around birth or soon after it. While the early maturation phase of the first primary molars takes place during the first year of life, late maturation of enamel takes several years [1,2].

Molar incisor hypomineralization (MIH) is a range of qualitative demarcated developmental enamel opacities that affect the first permanent molars either with or without involvement of the permanent incisors [3,4]. MIH etiology is multifactorial, involving several genetic, epigenetic and systemic factors that affect the transitional and maturation stages of amelogenesis during pregnancy and the first 3 years of life and result in hypomineralized, hypomatured enamel of normal thickness [5,6].

The term deciduous molar hypomineralization”(DMH) [3], also termed hypomineralized second primary molars (HSPM) [4], describes 1–4 s primary molars affected by hypomineralization.

The enamel of teeth with MIH has compromised physical characteristics. Compared with healthy teeth, hypomineralized defects have a lower mineral content, reduced hardness, increased porosity and increased content of carbonate and protein. The defects are asymmetrical and vary in severity from small opacities to severe defects with post eruption enamel breakdown that exposes the dentin [3,4,7]. As a result, teeth with MIH and DMH are more sensitive and have a higher prevalence of caries in comparison to healthy teeth [4,8]. The underlying mechanism for caries and increased sensitivity in teeth with MIH is not well understood. It has been suggested that it is caused by subclinical pulpal inflammation due to permeation of bacteria through the porous enamel [9] and anomalous pulpal innervation [10]. 

A relationship between prenatal conditions, such as smoking, consumption of alcohol, medications, maternal illness, and psychological stress and MIH has been reported. MIH was found to be significantly more prevalent among children whose mothers had problems during pregnancy than among children in the control group. Premature birth, prolonged delivery and Caesarian section, were the most common perinatal conditions associated with MIH [11,12].

MIH was also found to be associated with postnatal medical conditions, such as high fever [13], respiratory illness (bronchitis or asthma) in the first 4 years of life [14,15,16,17], otitis media [18,19], and chickenpox [20,21].

Antibiotics use in the postnatal period was also associated with MIH in several studies [13,14,18]; however, as antibiotics are usually given for upper respiratory tract infections, it was not confirmed whether the respiratory illness itself or the antibiotics was the cause of the association. A preclinical study in neonatal CD-1 mice showed that gentamycin and ampicillin given during tooth development reduced the proportion of enamel object volume and mineral density in first molars and incisors [22]. Exposure to environmental toxins, such as dioxins and bisphenol, was associated with MIH in several studies, but these associations remain controversial [23,24,25].

The relationship between MIH and socioeconomic factors, such as parents’ education or household annual income is disputed. A study from Brazil reported a higher risk for MIH among children in families with a high annual income [26]. Another study found a positive correlation between stress and the occurrence of MIH but not with the quality of family functioning [27].

Treatment of MIH is often challenging to both patients and dentists. The affected teeth can be sensitive to cold or heat. They tend to have enamel breakdown and to develop advanced caries lesions. Children with MIH endure greater dental treatment burden, including repeated and more frequent dental visits due to a high rate of failed restoration [28]. Children with severe MIH defects have worse oral health quality of life than unaffected children [29,30], which is probably due to its association with caries [31]. Therefore, to prevent this phenomenon, it is important to understand its prevalence and its causes. The aim of this study was to determine the prevalence of MIH and DMH among Israeli children and the risk factors contributing to this condition. 

## 2. Materials and Methods

### 2.1. Setting and Participants

This cross-sectional study was performed at the Department of Pediatric Dentistry at the School of Dentistry, Tel Aviv University (Tel Aviv, Israel) in 2019–2020. All patients aged 3 to 13 years who sought dental care at the Pediatric Clinic at the School of Dentistry, Tel Aviv University in 2019–2020 and had at least four erupted primary molars and/or four erupted permanent molars were offered a chance to participate in the study. Parents of children who were diagnosed with MIH and/or DMH were asked to complete a questionnaire. Children with syndromes related to enamel malformations, such as those with dental fluorosis, enamel hypoplasia or amelogenesis imperfecta and presence of orthodontic appliances, were excluded. Informed consent was obtained from the children’s parents or guardians after the purpose and procedures of the study were explained to them. The study was conducted in accordance with the Declaration of Helsinki and approved by the Ethics Committee of Tel Aviv University (protocol code 45.19, approval date 25 July 2019). 

### 2.2. Training 

Two pediatric dentists from the Department of Pediatric Dentistry performed all study evaluations. Prior to the study, they underwent theoretical training, which consisted of identifying MIH among 20 photographs of patients with MIH and 20 photographs showing other enamel defects.

### 2.3. Clinical Examination

The clinical visual examination was performed at the clinic using natural light. The child’s teeth were cleaned gently using gauze and were wet with saliva when examined. Mirrors were used for proper visualization and explorers were used to aid tactile sensation when needed. 

MIH was assessed using criteria based on the European Academy of Paediatric Dentistry (EAPD) 2003 guidelines [7], which included demarcated opacity, defined as a defect that changes the translucency of the enamel, variable in degree [32] and/or post-eruptive enamel breakdown, defined as a defect indicating mild to severe structure enamel loss after eruption of the tooth, e.g., hypomineralization-related attrition [33]. The defective enamel is of normal thickness with a smooth surface and can have white, yellow or brown opacities [34]. The demarcated opacity is not caused by caries, ingestion of excess fluoride during tooth development, amelogenesis imperfecta etc. All surfaces of the permanent first molar and the central incisor were examined for defined opacity. MIH was diagnosed when ≥1 permanent first molar was affected with or without the involvement of the incisors. DMH was diagnosed when ≥1 s primary molar was affected based on the criteria used by Elfrink et al. [35]. Enamel loss due to erosion and/or atypical restoration, whereby the size and form of the restoration do not match the present distribution of caries in the child’s mouth, e.g., amalgam, composite, glass ionomer and crown restorations [4], were excluded.

The severity of MIH and DMH was classified by the criteria delineated by Weerheijm et al. [7]. Mild lesions were those with demarcated opacities present in the non-stress-bearing areas of the molars without enamel loss from fracturing. Moderate lesions were lesions with any atypical restoration, demarcated opacities on the occlusal/incisal third of the teeth without post-eruptive enamel breakdown or with post-eruptive enamel breakdown limited to one or two surfaces with no involvement of the cusps. Severe lesions had post-eruptive enamel breakdown or atypical extractions [3]. The severity of MIH was defined by the most severe defect observed in the permanent first molars or permanent incisors. DMH severity was defined by the most severe defect seen in the primary second molars.

### 2.4. Questionnaire 

The questionnaire was based on one previously described by Koruyucu et al. [21]. It consisted of (1) demographic information (child’s age, sex and place of birth), (2) questions about the mother’s pregnancy (whether the mother was healthy or took any medications while she was pregnant, the delivery, whether there were any complications prior to or during childbirth and the child’s birth weight) and (3) the child’s medical history during the first three years of life.

### 2.5. Statistical Analysis

The data were analyzed by SPSS version 25 (IBM, SPSS Inc., Chicago, IL, USA) and by R software version 3.6.3 (https://www.r-project.org/, accessed on 18 November 2021). The data were summarized descriptively. To analyze the associations between demographic and clinical variables, continuous variables were tested using the Kruskal–Wallis test with Bonferroni corrections. Categorical variables were analyzed by chi-squared tests. Multivariate logistic regression was conducted to evaluate which of the significant variables found in the univariate analysis could predict a diagnosis of both MIH and DMH. A *p*-value < 0.05 was considered statistically significant.

## 3. Results

**Figure 1:**
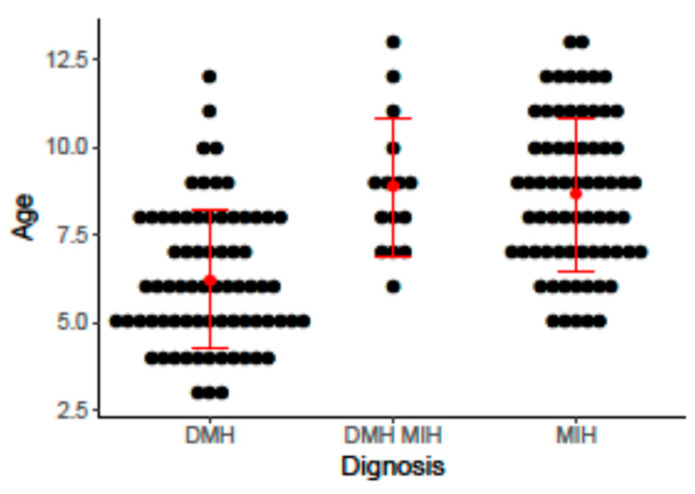
Distribution of hypomineralization in the study population by participant age. *p* < 0.01 for the difference among diagnosis groups. Abbreviations: MIH, molar incisor hypomineralization; DMH, deciduous molar hypomineralization.

A total of 1209 children with a median age of 5.5 years (range 3–13) were examined at the clinic. A total of 72 children (6.0%) were diagnosed with DMH, 68 children (10.33%) were diagnosed with MIH and 15 children (1.2%) were diagnosed with both DMH and MIH. Univariate analysis of the demographic variables, perinatal variables and variables related the children’s first 3 years of life showed that only MIH/DMH severity, the child’s age at MIH/DMH diagnosis and medications taken by the child’s mother during pregnancy were significantly different among the enamel defects type (Table 1). DMH was diagnosed at a statistically significantly mean younger age (Figure 1). Stratification by age showed that 43.7% of children ≤ 5 years were diagnosed with DMH, compared to 7.4% of children diagnosed with MIH; no children ≤ 5 years were diagnosed with both MIH and DMH (*p* < 0.01; Table 1). The percentage of children with MIH whose mother took any medication during pregnancy was statistically significantly lower compared to those with DMH or DMH + MIH (3.0% vs. 8.5% and 7.7%, respectively, *p* = 0.04; Table 1). A greater percentage of children had severe DMH and DMH + MIH compared to children with MIH alone (57.8% and 73.3% vs. 28.2%, respectively, *p* < 0.01; Table 1)

Multivariate logistic regression (Table 2) showed that age ≥ 5 years at diagnosis and severe hypomineralization significantly predicted a diagnosis of both DMH and MIH (χ^2^(3) = 11.08, *p* = 0.01), while it explained about 14.75% of total variance. The model was well-fit to the data (χ^2^(8) = 12.90, *p* = 0.11), as it classified about 90.20% of all observations. After adjusting for age, severity of hypomineralization was positively and significantly associated with a diagnosis of MIH + DMH, with an odds ratio of 4.18 (95% confidence interval [CI] 1.26–17.16), *p* = 0.03. 

## 4. Discussion

MIH prevalence in the study population was 10.3%, which is similar to that reported in Germany (10.1%) [36] and Greece (10.1%) [37] and is within the range reported by other studies conducted in various countries, including Northern Poland (6.4%) [38], Jeddah, Saudi Arabia (8.6%) [39], Iran (12.7%), India (13.1%), Northern Italy (13.7%) [40], Sweden (18.4%) [41], Iraq (18.6%) [28], Finland (19.3%) [42] and Brazil (19.9%) [43]. A very high prevalence of 40.2% was reported in another study conducted among Brazilian children. The range of MIH reported in the Middle East was 2.3–40.7%, with a mean prevalence of 15.1% [44]. In a study conducted among 413 adolescents aged 12 to 18 in a single town in Israel, 21.5% had MIH [45].

The DMH prevalence in our study population was 6.0%. The DMH prevalence reported for different countries ranged from 2.9% to 21.8%. The DMH prevalence reported in the current study was similar to that reported for the Netherlands [46], Iraq [28] and Saudi Arabia [47], but it was lower than that reported for Israeli adolescents aged 12–18 years [45]. Notably, a prevalence rate of >5% of any lesion is considered serious and has epidemiological significance. 

This difference in MIH prevalence may be attributed to the heterogeneity in ethnic and age groups studied, and it could also be explained by the differences in diagnostic criteria. There is no evidence in the literature associating ethnicity or race with MIH. In line with this, we did not find an association between being a Jewish or Arabic Israeli and the risk for MIH. The lack of association between race/ethnicity and MIH supports the assumption that MIH is a multifactorial condition involving the interaction between genetic vulnerability, epigenetic influence and exposure to systemic and environmental factors [48].

Since the development of the MIH criteria, observational studies have attempted to determine its etiological factors. A meta-analysis of 27 studies found that maternal psychological stress and illness, Caesarean section, delivery complications as well as respiratory disease and fever during the child’s first years of life were significantly linked with higher risk for MIH [6]. In a meta-analysis of 45 studies, perinatal factors leading to hypoxia, such as Caesarean section, prematurity and birth complications were correlated with greater risk for MIH. Postnatal factors, including gastric disorders, kidney diseases, urinary tract infection, otitis media, measles, pneumonia, bronchitis and asthma were also linked to MIH. Fever and use of antibiotics were also associated with MIH [5]. Another meta-analysis on studies conducted in Middle Eastern countries also found that pregnancy and early childhood illnesses and factors related to delivery were statically significantly associated with MIH [44]. Although prenatal, perinatal or early life illnesses or events have been associated with MIH, its underlying systemic causes have not been uncovered yet [8]. In the current study, we did not find an association between MIH and preterm birth, birth complications or low birth weight. In contrast, Elfrink et al. found that children with normal birth weight had lower risk for enamel defects in the primary dentition than those with low birth weight, which is possibly due to additional factors associated with maternal health status that increase the risk for enamel defects [49]. Our analysis showed that taking any medication during pregnancy was a risk factor for DMH or DMH + MIH compared to MIH.

Altogether, 1.24% of the children in the current study were diagnosed with both DMH and MIH. The first permanent molars develop during a period similar to that of second primary molars, with possible comparable risk factors for hypomineralization [50]. The appropriate age for diagnosing DMH is 3–5 years because the probability of diagnosing DMH is higher after all deciduous molars have already erupted [51,52]. Children with DMH are more susceptible to developing MIH. This relationship suggests a mutual cause and implies that DMH may be used as a predictor of MIH [4]. 

MIH and DMH severity differs among patients as well as within the same patient [3]. Our analysis showed that the severity of hypomineralization was a risk factor for being diagnosed with both DMH + MIH.

This study has several limitations. First, the study population included children who sought treatment at our clinic; therefore, it may not reflect the general population in Israel. Second, the differential diagnosis of MIH from early caries, enamel hypoplasia after trauma or amelogenesis imperfecta is difficult; thus, the prevalence of MIH and DMH may have been underestimated. Third, recall bias may have affected the parents’ answers regarding various variables during pregnancy and the child’s first years. 

## 5. Conclusions

The overall prevalence of MIH found in an academic pediatric dentistry clinic in Israel was 10.3%, and the overall prevalence of DMH was 6.0%, with no gender predilection; 1.2% of children were diagnosed with both MIH and DMH. Severe lesions confer a greater risk for having both MIH and DMH. Therefore, MIH should be diagnosed and monitored in young children to prevent further deterioration. Moreover, there is a need to plan and establish a program for preventing and restoring MIH and DMH among children.

As the current evidence for the etiology of MIH is lacking, longitudinal studies with a large sample size are needed to determine which factors contribute to the etiology of this condition. 

## Figures and Tables

**Table 1:** Participants’ demographics, perinatal clinical characteristics and MIH/DMH characteristics and association with having DMH, MIH or DMH + MIH.

	DMH N = 72	MIH N = 68	MIH + DMH N = 15	χ^2^ (df)	*p* Value
**Demographics**					
**Child’s sex, n (%)**					
Female	37 (52.1%)	29 (42.7%)	11 (73.3%)	4.86 (2)	0.09
Male	34 (47. 9%)	39 (57.4%)	4 (26.7%)		
**Child’s age at MIH/DMH diagnosis,** **years, mean (SD) [range]**	6.23 (1.97) [3,4,5,6,7,8,9,10,11,12]	8.65 (2.18) [5,6,7,8,9,10,11,12,13]	8.87 (1.96) [6,7,8,9,10,11,12,13]		<0.01
**Age categories, n (%)**					
≤5 years	31 (43.7%)	5 (7.4%)	0 (0%)	30.63 (2)	<0.01
>5 years	40 (56.3%)	63 (92.7%)	15 (100.0%)		
**Ethnicity, n (%)**					
Jewish	69 (97.2%)	62 (91.2%)	13 (86.7%)	3.34 (2)	0.19
Arab	2 (2.8%)	6 (8.8%)	2 (13.3%)		
**Parents’ place of residence, n (%)**					
Urban	68 (95.8%)	59 (86.8%)	13 (86.7%)	3.77 (2)	0.15
Rural	3 (4.2%)	9 (13.2%)	2 (13.3%)		
**Pregnancy characteristics**					
**Conception, n (%)**					
Spontaneous	68 (95.8%)	64 (94.1%)	12 (80.0%)	5.15 (2)	0.08
In vitro fertilization	3 (4.2%)	4 (5.9%)	3 (20.0%)		
**Illness during pregnancy, n (%)**	7 (9.9%)	3 (4.4%)	1 (6.7%)	1.56 (2)	0.46
**High-risk pregnancy, n (%)**	5 (7.0%)	6 (8.8%)	2 (13.3%)	0.66 (2)	0.72
**Any medication during the pregnancy, n (%)**	6 (8.5%)	2 (2.9%)	1 (7.7%)	1.94 (2)	0.04
**Delivery, n (%)**					
Regular	60 (84.5%)	50 (73.5%)	12 (80.0%)	3.95 (4)	0.41
Cesarean section	7 (9.8%)	13 (19.1%)	3 (20.0%)		
Vacuum	4 (5.6%)	5 (7.4%)	0		
**Analgesics during delivery, n (%)**					
Epidural	66 (93.0%)	61 (89.7%)	14 (93.3%)	1.82 (4)	0.77
Nitrous oxide	2 (2.8%)	1 (1.5%)	0		
None	3 (4.2%)	6 (8.8%)	1 (6.7%)		
**Gestational week at birth, n (%)**					
24–37	3 (4.2%)	5 (7.5%)	0	1.60 (2)	0.45
>37	68 (95.8%)	63 (92.7%)	15 (100.0%)		
**Birth weight, kg, n (%)**					
<1	1 (1.4%)	1 (1.5%)	0	1.70 (4)	0.79
1–2.5	5 (7.0%)	6 (8.8%)	0		
>2.5	65 (91.6%)	61 (89.7%)	15 (100.0%)		
**Child’s hospitalization, n (%)**					
During the first year of life	13 (18.3%)	6 (8.8%)	2 (13.3%)	2.65 (2)	0.26
During the second year of life	5 (7.0%)	4 (5.9%)	3 (20.0%)	3.51 (2)	0.17
During the third year of life	1 (1.4%)	4 (6.0%)	0	2.83 (2)	0.24
**Child’s illness, n (%)**					
During the first year of life	20 (28.2%)	12 (17.7%)	4 (26.7%)	2.25 (2)	0.32
During the second year of life	18 (25.4%)	10 (14.7%)	3 (20.0%)	2.45 (2)	0.29
During the third year of life	13 (18.3%)	11 (16.2%)	1 (6.7%)	1.23 (2)	0.54
**The child received antibiotics, n (%)**					
During the first year of life	35 (49.3%)	27 (39.7%)	7 (46.7%)	1.31 (2)	0.51
During the second year of life	24 (33.8%)	25 (36.8%)	7 (46.7%)	0.89 (2)	0.64
During the third year of life	19 (26.8%)	20 (29.4%)	5 (33.3%)	0.30 (2)	0.86
**The child had an ear infection, n (%)**					
During the first year of life	21 (29.6%)	17 (25.0%)	4 (26.7%)	0.37 (2)	0.83
During the second year of life	16 (22.5%)	14 (20.6%)	4 (26.7%)	0.28 (2)	0.87
During the third year of life	12 (16.9%)	10 (14.7%)	4 (26.7%)	1.25 (2)	0.53
**MIH/DMH severity, n (%)**					
Mild	30 (42.3%)	42 (61.8%)	4 (26.7%)	8.71 (2)	0.01
Severe	41 (57.8%)	26 (38.2%)	11 (73.3%)		

Abbreviations: df, degrees of freedom; DMH, deciduous molar hypomineralization; MIH, molar incisor hypomineralization; SD, standard deviation.

**Table 2:** Multivariate regression analysis for predicting MIH + DMH.

Variable	B	SE	Odds Ratio (95% CI)	*p* Value
Child’s age at diagnosis: >5 years (vs. <5 years)	0.35	0.14	1.42 (1.10–1.90)	0.01
Severity of hypomineralization: severe (vs. mild)	1.43	0.65	4.18 (1.26–17.16)	0.03
Medications during pregnancy: yes (vs. no)	0.59	1.14	1.80 (0.09–12.54)	0.60

Abbreviations: B, regression slope; CI, confidence interval; SE, standard error of the estimate.

## Data Availability

The data supporting the results are available from the corresponding authors upon reasonable request.
